# Sensory effects of COVID-19 in wine professionals

**DOI:** 10.1371/journal.pone.0321502

**Published:** 2025-04-25

**Authors:** Theodore J. Witek, Jr., Natasha Yasmin Sheikhan, Allan Tran

**Affiliations:** 1 Institute of Health Policy, Management, and Evaluation, University of Toronto, Toronto, Canada; 2 Dalla Lana School of Public Health, University of Toronto, Toronto, Canada; Bina Nusantara University, INDONESIA

## Abstract

**Objective:**

To evaluate the sensory impacts of COVID-19 infection among wine professionals, and consequences on personal and professional well-being. Our goal was to better understand these effects on an occupational cohort who relies heavily on intact sensory function.

**Design:**

The study employs an explanatory sequential mixed methods design comprising of two distinct phases: 1) a cross-sectional survey, followed by 2) qualitative interviews with a subsample of survey respondents.

**Setting:**

Wine professionals were recruited at the global level, excluding locations where Instagram is restricted or banned.

**Participants:**

Wine professionals (n=128) (ages ≥ 19 years) infected with COVID-19 and experienced sensory impacts were included in the study analysis. Eleven participants completed qualitative interviews.

**Interventions:**

None.

**Main outcome measures:**

Symptom profiles, details of taste and smell impact on personal and professional well-being. Effects on specific wine tasting attributes were also evaluated.

**Results:**

Infected participants reported typical COVID-19 symptoms. The most frequent first noticed symptoms were sore throat (21.09%; 27/128), loss of taste or smell (19.53%; 25/128), fever (17.19%; 22/128) and cough (16.41%; 21/128). For those infected and sensory affected, the extent of taste and smell loss was most reported as severe in the majority of cases. The duration of taste and smell loss was resolved within 4 weeks for most participants. A vast proportion of participants reported an impact on their involvement in the wine profession, with the impact severity ranging from significant (20.31%; 26/128), somewhat (57.03%; 73/128), and not at all (22.66%; 29/128). Additionally, participants predominately reported having some impact on their overall quality of life, which was characterized as severe (7.14%; 9/126), moderate (37.30%; 47/126), mild (30.16%; 38/126), and none (25.40%; 32/126).

**Conclusion:**

Wine professionals infected with COVID-19 and who experienced sensory alterations reported concerns about their professional and personal well-being with worries about a potential changing life narrative from losing vital sensory attributes. Policies to provide further resources, including therapeutics, for these professionals and others who suffer from sensory dysfunction are warranted.

## Introduction

Infection with COVID-19 typically manifests in a wide range of symptoms with episodes across the severity spectrum. Among the most common presenting symptoms are typical upper respiratory infection manifestations of cough, dyspnea, sore throat, nasal congestion and/or discharge as well as flu-like manifestations of fever/chills, fatigue, muscle ache headache, nausea and vomiting and/or diarrhea [1]. The development of new loss of taste and/or smell has emerged as a sentinel symptom [2] and the scale of this olfactory dysfunction observed in hundreds of millions of cases is unprecedented, even in previous pandemics [3].

The proportion of individuals infected with COVID-19 who experience these sensory effects varies depending on assessment methods, ranging in reports from 30% to 99% [2,4–8]. These sensory functions are vital in daily living (e.g., smelling danger, tasting spoiled food, etc.) and their absence or alteration affects the quality of daily living. The length of anosmia after COVID-19 can vary and persist for weeks [6,8,9], months [5,10] and even years [11–13]. For example, Tan et al. [13] reported persistent symptoms one year after hospitalization in COVID patients. Additionally in a one year follow up, Boscolo-Rizzo [11] noted persistence or worsening in 8.6% while McWilliams [12] noted persistence in 7.5% after 2 years.

The mechanism by which COVID-19 results in olfactory dysfunction is not entirely understood, but several attractive explanations exist. First, the presence of the viral receptor ACE2 in olfactory epithelium is likely associated with viral entry into the cell [3,11]. Butowt and colleagues [3] believe death of these cells supporting the mechanism of olfaction is associated with altered composition of mucus, and retraction of cilia on the olfactory receptor neurons, thus interfering with the olfactory signal transduction. They importantly note that this is consistent with both the rapid loss of COVID-19 induced anosmia as well as its rapid recovery when affected cells regenerate.

Wine professionals are a unique cohort related to sensory loss relative to the general population as they most often take a systematic approach to wine evaluation [14]. For example, there is a deliberate focus on primary (the grape and fermentation), secondary (post-fermentation developed aromas) and tertiary (maturation) aromas and flavors. The approach also includes evaluation of sweetness, acidity, tannin, alcohol, body, and finish [14]. They are often highly proficient in identifying sensory characteristics of wine on the nose and palate and these senses are a fundamental requirement for their livelihood.

The purpose of this study was to evaluate the impact of sensory dysfunction in a convenience sample of wine professionals. We chose this occupational cohort given their relative expertise in sensory evaluation and reliance of intact sensory function for their livelihood.

## Methods

### Study design

The present study adopted an explanatory sequential mixed methods design. There were two distinct phases: 1) a cross-sectional survey and 2) qualitative interviews with a subsample of survey respondents from the previous phase. An explanatory sequential design was chosen such that the qualitative interview data could provide greater insight and depth into the initial quantitative results [15]. Both phases of the study were approved by the University of Toronto Research Ethics Board (REB). The REB approval was granted from 06/12/2022–05/12/2024, with a renewal made during this period. The recruitment period for the survey was from 06/12/2022 through the completion of the last qualitative interview on 13/09/2023, with the first survey response recorded on 19/12/2022. Qualitative interviews were conducted within the REB-approved timeframe, from 07/05/2023–13/09/2023.

### Phase 1: quantitative strand

#### Survey development and standardization.

In Phase 1, an expert advisory group made up of 5 senior wine professionals with lived experience of COVID-19 infection were formed to improve the relevance of the questionnaire. Advisory group members discussed what they have observed related to a COVID-19 infection within their professional wine activity. These individuals pilot-tested the questionnaire flow and function, leading to minor revisions of the final survey questionnaire.

The study utilized a web-based application Research Electronic Data Capture (REDCap), to build and manage our online survey which served as a database for the collection of the quantitative data. REDCap is designed for secure storage of confidential information and offers an efficient workflow for collecting and exporting the data to analytical software and statistical programs.

#### Data collection.

Phase 1 included a cross-sectional survey with a convenience sample of wine professionals. The advisory group members, in addition to other individuals well-connected in the wine profession, served as promoters for snowball recruitment through their wine professional contacts and circulated the survey link to their professional networks. Additionally, advertisements were placed on Instagram for open recruitment. Any individual who was infected with COVID-19 was invited to participate knowing this was a study on the sensory effects of COVID-19 in wine professionals.

#### Data analysis procedure.

The analyses in Phase 1 were conducted using SAS^®^ OnDemand for Academics [16]. The descriptive results (frequencies and percentages) were computed to examine the distribution of characteristics among individuals who had COVID-19 infection and sensory impacts. For some results, the denominator for the total number of participants infected with COVID-19 and had sensory impacts was reduced due to missing responses.

### Phase 2: qualitative strand

#### Data collection.

To further understand the impact of a COVID-19 infection on wine professionals, semi-structured interviews were conducted from a pool of respondents in the Phase 1 survey. Specifically, we aimed to capture great depth of the professionals’ lived experiences from being infected with COVID-19 and being sensory affected as a wine professional.

At the end of the Phase 1 questionnaire, all 128 participants that reported an impact on taste or smell were sent an email invitation to participate in the qualitative review. Twelve participants who were COVID-19 infected and sensory affected agreed to participate in the qualitative interviews, of which 11 followed through. Following Phase 1, an interview guide was developed by the research team and received ethical approval. The interview guide included 20 questions/sub-questions and was pilot tested with members of the expert advisory group to increase relevance and rigor.

Qualitative interviews were conducted by the first author (TJW) via Zoom, a teleconference platform, and informed consent was obtained verbally. Interviews were conducted from 07/05/2023–13/09/2023 and lasted between 13.54 to 25.38 minutes. Interviews were audio-recorded, transcribed, and anonymized.

#### Data analysis procedure.

Underpinned by a pragmatist epistemology, the qualitative data were analyzed inductively following Braun and Clarke’s reflexive thematic analysis [17]. Reflexive thematic analysis occurred in 6 iterative phases: familiarization with the data, generating the initial codes, searching for themes/subthemes, reviewing and mapping out themes/subthemes, defining themes and extracting quotes, and writing up the findings. Themes were defined as patterns of meaning. Data were coded by the first author (TJW) and conducted at both the semantic and latent levels. Team debriefings were used during the analysis to enhance credibility; for instance, TJW met with NYS to refine codes, merge codes, and discuss themes development.

## Results

### Quantitative results

#### Respondent characteristics.

The survey was completed by 199 individuals who were infected by COVID-19. Of these 199 infected individuals, 128 individuals (128/199; 64.32%) reported impacts on their taste or smell. Patient characteristics, wine industry qualifications, and experience are detailed in Table 1. Additionally, a large majority of respondents had their primary source of income derived from the wine industry and/or hospitality (116/128; 90.63%) (Table 1).

**Table 1:** Participant Demographics (Infected and Affected).

Characteristics	Participants, (n, %)
**Gender**	
*Man*	54 (42.86)
*Woman*	71 (56.35)
*Other*	1 (0.79)
**Age**	
*19–29 years old*	15 (11.72)
*30–39 years old*	54 (42.19)
*40–49 years old*	35 (27.34)
*50*+ *years old*	24 (18.75)
**Country**	
*United States*	39 (30.47)
*Canada*	52 (40.63)
*New Zealand*	3 (2.34)
*United Kingdom*	8 (6.25)
*Australia*	1 (0.78)
*France*	3 (2.34)
*Portugal*	17 (13.28)
*Spain*	2 (1.56)
*United Arab Emirates*	1 (0.78)
*Mexico*	1 (0.78)
*Not Specified*	1 (0.78)
**Ethnicity**	
*Asian*	11 (8.59)
*Black*	13 (10.16)
*Indigenous*	4 (3.13)
*Latin American*	5 (3.91)
*Mixed heritage*	5 (3.91)
*White (North American and European)*	94 (73.44)
*Other*	1 (0.78)
**Qualification**	
*High-level formal qualification* ^*^	49 (38.28)
*Low-mid level qualification*	56 (43.75)
*No formal qualification*	23 (17.97)
**Experience in wine industry**	
≤*5 Years*	41 (32.03)
*>5 Years* ≤*10 Years*	42 (32.81)
*>10 Years*	45 (35.16)
**Primary income from wine industry**	
*Yes*	116 (90.63)
*No*	12 (9.38)

* *MW, Masters of Wine or WSET Diploma, Wine & Spirit Education Trust.*

### COVID-19 infection and overall symptoms

Among the total participants who had sensory impacts (n=128), 54 individuals (54/125; 43.20%) indicated having more than one COVID-19 infection. The first episode was used in the survey. Method of infection, detection, and vaccination status are listed in Table 2.

**Table 2:** Background data of participants (infected and affected).

**Characteristics**	**Participants, (n, %)**
**Documented infection with sensory impacts (smell or taste)**	128 (100.00)
**More than 1 COVID-19 infection**	
*Yes*	54 (43.20)
*No*	71 (56.80)
**Experienced symptoms**	
*Yes*	122 (97.60)
*No*	3 (2.40)
**Method of diagnosis**	
*Rapid Antigen Test*	60 (47.24)
*PCR Test*	28 (22.05)
*Both*	38 (29.92)
*Neither*	1 (0.79)
**Period of initial infection**	
*Sometime during 2020*	24 (18.90)
*Sometime during 2021*	52 (40.94)
*Sometime during 2022*	51 (40.16)
**COVID-19 treatment by HCP**	
*Yes*	48 (37.50)
*No*	80 (62.50)
**COVID-19 symptom severity**	
*Mild*	28 (22.05)
*Moderate*	80 (62.99)
*Severe*	19 (14.96)
*Hospitalized*	
*Yes*	22 (17.46)
*No*	104 (82.54)
**First noticed COVID-19 symptoms**	
*Cough*	21 (16.41)
*Fever*	22 (17.19)
*Headache*	11 (8.59)
*Aches and pains*	7 (5.47)
*Loss of taste or smell*	25 (19.53)
*Sore throat*	27 (21.09)
*Tiredness*	14 (10.94)
*Other*	1 (0.78)
COVID-19 symptoms over the course of acute illness period*	
*Fever*	91 (71.09)
*Cough*	84 (65.63)
*Tiredness*	92 (71.88)
*Loss of taste*	82 (64.06)
*Loss of smell*	88 (68.75)
*Sore throat*	69 (53.91)
*Headache*	72 (56.25)
*Aches and pains*	72 (56.25)
*Other*	12 (9.38)
**Attempt to retrain senses**	
*Yes*	83 (64.84)
*No*	45 (35.16)
**Success in training senses**	
*Successful*	31 (38.27)
*Partially successful*	38 (46.91)
*Unsuccessful*	12 (14.81)
**Experienced prolonged COVID symptoms**	
*Yes*	66 (51.97)
*No*	61 (48.03)
**Duration of COVID-19 symptoms**	
*Less than a month*	30 (45.45)
*Greater than 1 month and less than 6 months*	23 (34.85)
*Greater than 6 months*	13 (19.70)
**Expressed worries about the potential effects on your ability to taste and identify** **wine**	
*Very worried*	61 (47.66)
*Somewhat worried*	56 (43.75)
*Not at all worried*	11 (8.59)
**Discussed the potential of losing your taste and/or smell with a colleague or** **friend/family**	
*Yes*	110 (86.61)
*No*	17 (13.39)
**Vaccination status**	
*Vaccinated*	124 (98.41)
*Unvaccinated*	2 (1.59)

*PCR, polymerase chain reaction; HCP, healthcare provider*

**Fig 1:**
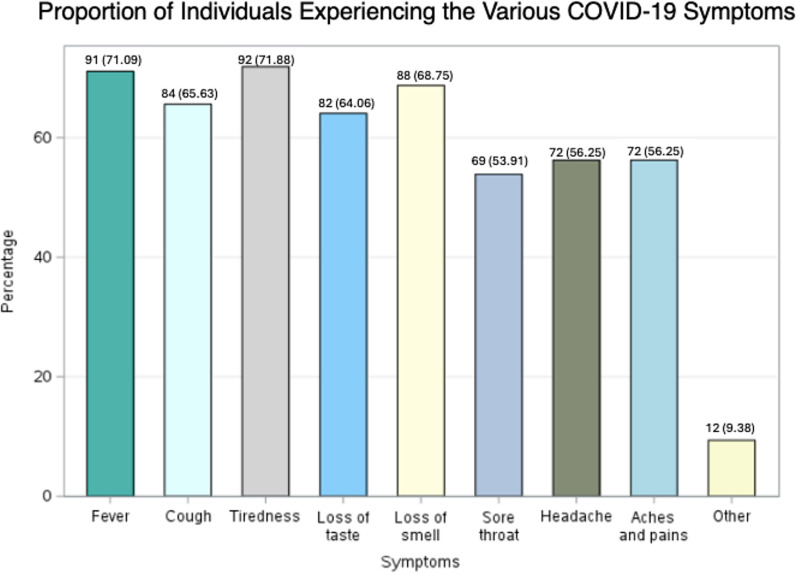
Proportion of Individuals Experiencing the Various COVID-19 Symptoms.

The majority of respondents to the initial survey had experienced COVID-19 symptoms (122/125; 97.60%) with the majority reporting their severity as moderate (80/125; 62.99%). The first noticed COVID-19 symptoms were sore throat (21.09%), loss of taste or smell (19.53%), fever (17.19%), cough (16.41%), tiredness (10.94), headache (8.59%), aches and pains (5.47%), and other (0.78%). The proportion of patients experiencing the various COVID-19 symptoms is presented, with over half of the respondents experienced each of the hallmark symptoms of COVID-19 infection (Fig 1). Over one-third of the respondents sought care from a healthcare professional (48/128; 37.50%). Additionally, 22 individuals (22/126; 17.46%) were hospitalized Table 2.

### Long-term COVID-19 symptoms

Among those who self-reported experiencing prolonged COVID-19 symptoms following infection (66/127; 51.97%), 13 individuals (13/66; 19.70%) experienced their symptoms for more than 6 months at the time of the survey completion Table 2.

### Sensory effects & attempts to alleviate sensory effects

Of the total respondents with COVID-19 infection and sensory impacts (n=128), 61 individuals (61/128; 47.66%) were very worried about the potential effects of COVID-19 infection on their ability to taste and identify wine, 56 individuals (56/128; 43.75%) were somewhat worried, and 11 individuals (11/128; 8.59%) were not at all worried. Furthermore, 110 respondents (110/127; 86.61%) discussed the potential of losing their taste and/or smell with a colleague, friend, or family.

Out of the 128 individuals infected with COVID-19 and had sensory impacts, 83 respondents (83/128; 64.84%) attempted to retrain their senses. Among those who attempted to retrain their senses and reported their success (n=81), 31 individuals (31/81; 38.27%) reported successful, 38 individuals (38/81; 46.91%) reported partially successful and 12 individuals (12/81; 14.81%) reported being unsuccessful.

### Effects on taste

Within the cohort of participants experiencing COVID-19 infection (n=199) and reported effects on taste, 69 individuals (69/199; 34.67%) experienced loss of taste, 44 individuals (44/199; 22.11%) had a distorted sense of taste, and 86 individuals (86/199; 43.22%) had no changes to taste. For those who reported a loss of taste and its severity (n=67), they graded the loss as mild (10/67; 14.93%), moderate (25/67; 37.31%), and severe (32/67; 47.76%). Among the 44 individuals who reported a distorted sense of taste and its severity (44/199; 22.11%), the distortion was categorized as mild in 25 individuals (25/44; 56.82%), moderate in 16 individuals (16/44; 36.36%), and severe in 3 individuals (3/44; 6.82%). For those who either had a loss of taste or taste distortion (n=111), most respondents experienced these symptoms for more than 1 week but less than 1 month (62/111; 55.86%). However, among those with symptoms greater than 6 months (n=7), 5 individuals (5/111; 4.46%) still had persisting symptoms at time of completing the questionnaire. Additionally, 101 individuals (101/112; 90.18%) who experienced taste loss or distortion discussed their concerns with a colleague, friend, or family Table 3.

**Table 3:** Participants experience with taste and smell.

Characteristics	Participants (n, %)	
**Participants with COVID-19 infection**	199 128 (64.32)	
**COVID-19 infected participants with impacts on either taste or smell**		
	**Taste**	**Smell**
**Sensory changes**	113 (56.78)	112 (56.57)
*Loss of taste or smell*	69 (34.67)	75 (37.88)
*Distorted taste or smell*	44 (22.11)	37 (18.69)
*No changes to taste or smell*	86 (43.22)	86 (43.43)
*Extent of loss*	*(n* ***=*** *67)*	*(n* ***=*** *74)*
*Severe/ pronounced reduction (e.g., I could not taste/smell* *anything)*	32 (47.76)	36 (48.65)
*Moderate reduction (e.g., I lost most but not all of my taste/smell)*	25 (37.31)	25 (33.78)
*Mild/ minor, e.g., I lost a bit of my sense to* *taste/smell)*	10 (14.93)	13 (17.57)
*Extent of distortion*	*(n* ***=*** *44)*	*(n* ***=*** *37)*
*Severe/ pronounced reduction (e.g., My taste/smell was* *completely distorted)*	3 (6.82)	7 (18.92)
*Moderate reduction (e.g., My taste/smell was semi-distorted)*	16 (36.36)	14 (37.84)
*Mild/ minor reduction (e.g., My taste/smell was a bit* *distorted)*	25 (56.82)	16 (43.24)
*Duration of loss*	*(n* ***=*** *111)*	*(n* ***=*** *112)*
*About 1 week*	25 (22.52)	26 (23.21)
*Greater than 1 week and less than 1 month*	62 (55.86)	50 (44.64)
*Greater than 1 month and less than 6 months*	17 (15.32)	29 (25.89)
*Greater than 6 months*	7 (6.31)	7 (6.25)
*Symptoms still persisting*	*(n* ***=*** *111)*	*(n* ***=*** *112)*
*Yes*	5 (4.46)	5 (4.46)
*No*	2 (1.80)	2 (1.79)
*Discussed concern about losing your taste/smell or experiencing* *taste/smell distortion with a colleague, friend or family*	*(n* ***=*** *112)*	*(n* ***=*** *109)*
*Yes*	101 (90.18)	96 (88.07)
*No*	11 (9.82)	13 (11.93)
**Experience changes in both sense of smell and taste**	98 (49.25)	
**Participants with no impacts on both taste and smell**	71 (35.68)	
*Feelings about potential effects on wine profession (n* ***=*** *70)*		
*Very worried about any potential effects on smell or taste on your* *wine profession*	12 (17.14)	
*Slightly worried about any potential effects on smell or taste on* *your wine profession*	29 (41.43)	
*Not worried about any potential effects on smell or taste on your* *wine profession*	29 (41.43)	
*Discussed your concern about potentially losing your taste or smell* *from* ***COVID****-19 infection with a colleague or friend, or family (n****=****71)*		
*Yes*	43 (60.56)	
*No*	28 (39.44)	

*The denominators may vary and be less than the total sample size due to missing data for some responses.*

### Effects on smell

Among those with COVID-19 infection (n=199) and reported effects on smell, 75 individuals (75/198; 37.88%) had a loss of smell, 37 individuals (37/198; 18.69%) had a distortion in smell, and 86 individuals (86/198; 43.43%) had no changes in smell. Of those that reported a loss of smell and its severity (n=74), 13 individuals (13/74; 17.57%) expressed their experience as mild, 25 individuals (25/74; 33.78%) as moderate, and 36 individuals (36/74; 48.65%) as severe. Among the 37 individuals (37/199; 18.59%) who reported a distorted sense of smell and its severity, the distortion was categorized as mild in 16 individuals (16/37; 43.24%), moderate in 14 individuals (14/37; 37.84%), and severe in 7 individuals (7/37; 18.92%). Of those individuals experiencing loss of smell or smell distortion (n=112), most respondents experienced these symptoms for more than 1 week but less than 1 month (50/112; 44.64%). 7 individuals (7/112; 6.25%) had symptoms greater than 6 months, and 5 individuals (5/112; 4.46%) had persisting symptoms at the end of the study period. Finally, 96 individuals (96/109; 88.07%) who experienced a loss of smell or smell distortion discussed their concerns with a colleague, friend, or family.

### Effects on taste and smell

Among the individuals infected with COVID-19 (n=199), 98 (98/199; 49.25%) respondents experienced changes in both sense of taste and smell Table 3.

### No effects on taste and smell

From the total of 199 individuals with COVID-19 infection, 71 respondents (71/199; 35.68%) who reported not having any impacts on taste and smell expressed their feelings about potential effects on their wine profession. 29 individuals (29/70; 41.43%) were not worried, 29 individuals (29/70; 41.43%) were slightly worried, and 12 individuals (12/70; 17.14%) were very worried Table 3. Of these individuals without impacts on their taste and smell (n=71), 43 respondents (43/71; 60.56%) discussed their concerns about potentially losing their taste or smell from COVID-19 infection with a colleague, friend, or family.

### Impact on daily living

Among the 128 individuals with COVID-19 infection and were sensory affected, respondents reported their impacts on their wine profession. 26 individuals (26/128; 20.31%) experienced a significant impact, 73 individuals (73/128; 57.03%) experienced a moderate impact, and 29 individuals (29/128; 22.66%) had no impact at all. Of those infected and sensory affected (n=128), 44 individuals (44/128; 34.38%) required time away from their profession. Finally, individuals reported having impacts on their overall quality of life ranging from severe (9/126; 7.14%), moderate (47/126; 37.30%), minor (38/126; 30.16%) to none (32/126; 25.40%) Table 4.

**Table 4:** Impact on Professional Life of Participants (n=128) (Infected and Affected).

Characteristics	Participants, (n, %)
**Participants with impacts on taste or smell**	
Impact on involvement with wine profession	
*Significantly*	26 (20.31)
*Somewhat*	73 (57.03)
*Not at all*	29 (22.66)
*Require time away from profession*	
*Yes*	44 (34.38)
*No*	84 (65.63)
Impact on your overall quality of life	
*Severe*	9 (7.14)
*Moderate*	47 (37.30)
*Minor*	38 (30.16)
*None*	32 (25.40)

### Specific wine attributes

**Fig 2:**
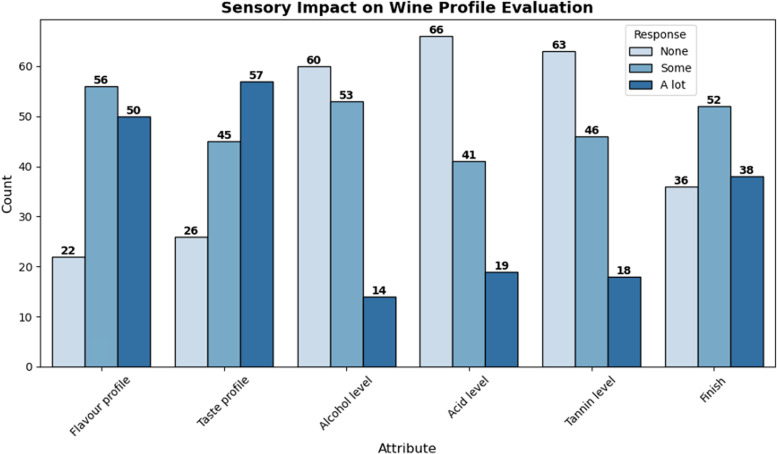
Sensory Impact on Wine Profile Evaluation.

Individuals with COVID-19 infection and were sensory affected (n=128), reported their impacts on specific wine attributes as a lot, some, or none. For flavour profile: a lot (50/128; 39.06%), some (56/128; 43.75%), none (22/128; 17.19%); taste profile: a lot (57/128; 44.53%), some (45/128; 35.16%), none (26/128; 20.31%); alcohol level: a lot (14/128; 10.94%), some (53/128; 41.41%), none (60/128; 46.88%); acid level: a lot (19/128; 14.84%), some (41/128; 32.03%), none (66/128; 51.56%); tannin level: a lot (18/128; 14.06%), some (46/128; 35.94%), none (63/128; 49.22%); and finish: a lot (38/128; 29.69%), some (52/128; 40.63%), none (36/128; 28.13%) (Fig 2).

### Qualitative results

To gain a deeper understanding of wine professionals infected with COVID-19 and sensory affected, 11 survey respondents from Phase 1 participated in qualitative interviews. The following three main themes, each with their respective subthemes, were constructed from the data: (1) initial early experience with infection; (2) ways of coping and reactions to illness; and (3) negative impact on personal and professional life.

#### Theme I: Initial early experience with infection.

##### Subtheme 1: Moment of realization.

When reflecting on their early experiences with a COVID-19 infection, wine professionals described the moment of realization, fears around the progression of illness, and their experiences with anosmia. The moment of realization ranged from mild flu-like symptoms to more severe emotional and physical experiences. For instance, one participant described it as *“Never forget the moment because it completely changed my life…”* (Participant 3). Anosmia, for some, demarcated the moment of realization: *“Then it came out [that loss of taste/smell] was an indicator you may have [COVID-19]* (Participant 6).

##### Subtheme 2: Fears around progression of illness

As wine professionals realized they had COVID-19, many developed fears about the progression of the illness, especially as it pertained to anosmia and the impact it may have on their careers in the wine industry. For instance, one participant stated: *“I feel like oh my god, oh my god it’s going to be something again…I’m going to lose my palate”* (Participant 7). Several experienced feelings of uncertainty, especially around the ‘unknown’ symptom progression, not knowing when they would be able to return to work, and fears around whether they would even recover from anosmia. For instance, one described having a *“momentary panic because you hear stories of people who have not recovered for six months down the line”* (Participant 1).

##### Subtheme 3: Negative experiences with changes in smell or taste

All participants described their experiences with anosmia. Many described the initial shock they experienced as their taste and smell palette either changed or disappeared; for instance, one participant recalled their initial thought process after tasting wine for the first time as: *“Why the f—k does this taste like petrol—like really weird. Is there something wrong with the glass?”* (Participant 3)*.* Others shared similar experiences with wine tasting bad, comparing the taste to ‘corked wine’, ‘burnt smell’, and an unpleasant experience. One used sand as a way to describe their experience drinking wine: *“ My mouth felt like filled with sand all the time…my throat was dry and covered in sand […] [wine] smell like burnt sand.”* (Participant 4). Participants who lost their sense of taste compared wine to tasting like water, ‘muted’, and simply ‘nothing’. Similar to their initial fears around the progression of illness, many described their initial fears after they lost their palette as a frustrating, horrifying, and panic-ridden experience.

#### Theme II: Ways Of coping/reactions to illness.

##### Subtheme 1: Retraining one’s nose/remedies

As wine professionals reflected on their journey with anosmia, they described several ways of retraining their noses. This included a range of methods such as holistic remedies, practice kits, and stepped training. Holistic remedies included taking magnesium, in addition to smelling essential oils, tabasco, coffee, lavender salts, pepper, eucalyptus, ginger, and burning oranges. Others described going through a stepped re-training process, such as: *“I started to try a little bit of wine. Was a bit more selective…more acidic? More aromatic? Went to extremes, aromatic & taste profile”* (Participant 2).

##### Subtheme 2: Information seeking, sharing, and support

As wine professionals dealt with anosmia, many sought information on re-training their noses through a variety of venues. Several included seeking information from the internet (e.g., Google) and their colleagues in the wine industry; a few also sought information from their physicians and social media. In addition to seeking information from other wine professionals/colleagues, many shared their frustrations with their colleagues. This also helped participants develop a stronger sense of community and emotional support system from their colleagues: *“I went to my little tight group of sommeliers that I taste with. Everyone was like more an emotional support like don’t worry it could happen to multiple of them”* (Participant 11). Interestingly, a few wine professionals felt understood by chefs who underwent a similar experience with anosmia, with some even seeking information and advice from chefs who had experienced anosmia. For example, *“Chefs were the only persons who realized what I was going through…not getting things back. They were really concerned for me.”* (Participant 4).

##### Sub-theme 3: Experience with recovery

For participants who lost their sense of taste and/or smell, several described stronger scents as eventually reigniting their senses, such as citruses, pungent scents (*e.g.,* skunks, manure), and stronger herbs: *“If there was a unifying factor which made things through it would be like high toned and very pungent so resonance herbs like rosemary and mint.”* (Participant 1). As they regained their ability to smell, many described a sense of hope and excitement during this process. Many described ways of coping by finding ways to stay connected to the wine industry: *“Went to many wine fairs in many countries…only thing that kept me going…I had to go-to keep connected to the world”* (Participant 3).

#### Theme III: Negative impact on daily life/work life.

##### Subtheme 1: Negative impact on experience with wine/ wine tasting

Most participants described the negative impact of COVID-19 on their experience with wine and wine tasting. This included changes in their ability to distinguish between different types of wine, which ranged from a partial inability to distinguish between wines to a complete inability to distinguish between wines. For instance, one participant noted, “*I couldn’t have told you if it was red or white based on smell”* (Participant 11). Several participants noted that they had to rely on their prior knowledge of what wine tastes like during wine tastings and how other professionals describe the wine. Some described negative impacts on their sensory perception of specific wine attributes, explaining a range of differences in their ability to perceive tannins, acidity, and tasting notes: *“I could still perceive tannin in the sense that I could feel my mouth had dried. I could feel the physical sense effect but tannin tasted particularly bitter.”* (Participant 1). Participants described the shift in their tasting experience as distressing, confusing, and strange.

##### Subtheme 2: Negative impact on mental health

As participants experienced shifts in their ability to taste and smell wine, they reported a negative impact on their mental health, with several recalling the initial shock and panic when they first experienced a loss in their senses. The prolonged effect on their inability to taste led to experiences of anxiety, depression, and an overall negative impact on their well-being: *“I couldn’t sleep…I was stressed …I had anxiety. […] I was severely depressed for a couple of weeks until it started to come back”* (Participant 10)

##### Subtheme 3: Negative impact on career

With shifts in participants’ ability to assess wine, many described a negative impact on their wine career. This included a negative impact on their performance; for instance, some describing that they would ‘work blind’ and felt ‘sensory overload’ in the workplace. Many described the chronic stress they experienced around their ability to succeed in the wine industry. This also included second-guessing whether they should switch careers, and if their wine career was over:

*“We all love wine, we all love food, and to have to have that taken away from me is unimaginable. And you know, the last ten years of my life I’ve dedicated to wine. But...to be faced with the possibility that I would never really be able to work in wine again…”* (Participant 8).

In contrast, one described being a better sommelier as they had to work harder as they retrained their nose.

The insights from the integration of qualitative and quantitative findings are summarized in Table 5. It is evident that the qualitative findings expanded on several aspects of the quantitative survey, with many providing real-world impact of the data observed.

**Table 5:** Joint display of qualitative and quantitative findings.

Qualitative Findings	Relevant Quantitative Findings	Integration Insights
**Theme 1: Initial early experience with infection**		
Subtheme 1: Moment of realization “Never forget the moment, because it completely changed my life…” (Participant 3)	• 19.5% reported loss of taste or smell as the first symptom • 34.7% experienced taste loss and 22.1% experienced taste distortion • 37.9% experienced smell loss and 18.7% experienced smell distortion	Qualitative findings expand on quantitative symptom profile and provide a vivid description of participants’ experiences with sensory disruptions. Notably, early sensory loss was a pivotal moment for participants.
Subtheme 2: Fears around progression of illness “I feel like oh my god, oh my god it’s going to be something again…I’m going to lose my palate” (Participant 7)	• 47.7% felt very worried and 43.8% felt somewhat worried about potential sensory effects on wine profession	Qualitative findings expand on the high levels of worry around career progression and professional identity, including the fears of not recovering sensory abilities and career loss.
Subtheme 3: Negative experience with sensory loss “[wine] smelled like burnt sand.” (Participant 4).	• 85.1% experienced moderate or severe taste loss • 43.2% experienced moderate or severe taste distortion • 82.4% experienced moderate or severe smell loss • 56.8% experienced moderate or severe smell distortion	Qualitative findings expand on the severity of sensory effects, detailing their experiences with sensory loss and distortion (e.g., pungent and unpleasant tastes).
**Theme 2: Ways of coping and reacting to illness**		
Sub-theme 1: Retraining one’s nose/remedies “I started to try a little bit of wine. Was a bit more selective...” (Participant 2)	• 64.8% attempted to retrain their senses	Qualitative findings explain the specific approaches that participants underwent to retrain their noses, including proactive coping mechanisms.
Subtheme 2: Information seeking, sharing, and support “I went to my little tight group of sommeliers that I taste with...” (Participant 11)	• 90.2% discussed concern about losing their taste or taste distortion with a colleague, friend or family • 88.1% discussed concern about losing their smell or smell distortion with a colleague, friend or family	Qualitative findings expand on information seeking strategies and community of supports formed as a result of sensory disruptions.
Subtheme 3: Experience with recovery “Went to many wine fairs in many countries…only thing that kept me going…” (Participant 3)	• 6.3% experienced a loss or distortion of taste for over 6 months • 6.3% experienced a loss or distortion of smell for over 6 months	Qualitative findings detail participants’ experiences with prolonged symptoms and uncertainty, including the personal and professional impact on the long-term effects of sensory disruptions.
**Theme 3: Negative impact on daily life and work life**		
Subtheme 1: Negative impact on experience with wine and wine tasting I could feel the physical sense effect, but tannin tasted particularly bitter.” (Participant 1)	• 82.8% impact on evaluating some to a lot of flavour profile • 79.7% impact on evaluating some to a lot of taste profile • 52.4% impact on evaluating some to a lot of alcohol level • 46.9% impact on evaluating some to a lot of acid level • 50.0% impact on evaluating some to a lot of tannin level • 70.3% impact on evaluating some to a lot of finish level	Qualitative findings expand on the wine evaluation profile, detailing participants’ personal experiences with the negative impact on their work.
Sub-theme 2: Negative impact on mental health “I couldn’t sleep…I was stressed …I had anxiety...” (Participant 10)	• 44.4% experienced moderate or severe impact on quality of life	Qualitative findings explain the negative impact on quality of life and provide insights on the impact on mental health that are rooted in participants’ fear around their profession and ability to perform.
Sub-theme 3: Negative impact on career “To be faced with the possibility that I would never really be able to work in wine again…” (Participant 8).	• 20.3% experienced a significantly negative impact on their involvement with the wine profession • 34.4% required taking time away from the profession	Qualitative findings expand on the negative impact on their career, including the inability to perform core sensory tasks.

**Note:** several categories have been aggregated in this table for brevity.

## Discussion

The global wine industry employs over 1 million [18] and plays a significant economic role, with annual sales projected to be USD 457 billion by 2028 [19]. Wine professionals in the hospitality industry often receive years of training and utilize their sensory skills in a systematic approach to wine assessment. We evaluated the impact of COVID-19-induced anosmia and ageusia on wine professionals. The quantitative findings highlighted the impacts on personal and professional well-being. Building on the quantitative findings, the qualitative findings provide a deeper understanding of the emotional and professional toll on individuals whose careers and identities are intertwined with their sensory abilities.

Symptom profiles were typical of COVID-19 infection with sensory impacts among the most common presenting symptoms in this cohort. Like with the common cold [20], sore throat was also a frequent first-noticed symptom in several participants, but unlike other acute respiratory infections, sensory effects were noticed first by many, thus reinforcing it as a signal symptom for COVID-19 infection [2]. The sensory losses described by participants were the foundation of stress and fear related to their profession and love of wine.

COVID-19 infection was objectively documented in nearly all participants with most infections occurring in 2021 and 2022. Most suffered moderate symptoms with over one-third of them seeking care from a healthcare practitioner and 17% requiring hospitalization. The time of the reported infection during the pandemic has relevance. For example, a case-control study [21] found the prevalence of olfactory dysfunction during the wave of the Omicron variant to be markedly lower than that of the D614G, Alpha and Delta variants. Thus, more impact could have been expected if more respondents suffered from infections caused by earlier variants.

Our participants were a convenient sample that were well characterized in terms of professional status. Formal qualifications were reported in 82% of participants with 65% having more than five years of experience. Also, over 90% of the respondents reporting that their primary income was derived from the wine industry. As such, this supports a study cohort that would take a systematic approach to tasting wine with refined sensory skills [14].

The impacts on professional life were prominent with moderate or severe impacts reported in over three-quarters of participants. The requirement for intact senses for professional functioning was evident in the interviews. For example, all participants noted absence or alteration in taste and/or smell in the context of their approaches in assessing wine for their occupation. Concern for one’s professional future was common.

The fact that most participants were very worried about COVID-19 infection affecting their smell and taste with most discussing this concerns with friends and family indicate the psychosocial burden in this particular occupational cohort. Even in those infected that were not sensory affected, the majority of participants expressed some degree of worry with the majority also discussing their concerns with friends, family or colleague.

Career satisfaction is an important variable in one’s quality of life [22]. This manifested during the COVID-19 pandemic as reported by Eicher et al. [23] in the CORONA HEALTH app study in Germany. They reported that job seekers or those who saw their work hours reduced or who could not pursue their regular jobs presented a lower quality of life in individual areas of life than the respective reference group. While these general findings may reflect negative effects from financial burdens or limitations in social interaction opportunities, we believe that the specific COVID-induced effects reported by wine professionals contributed to a unique aspect of stress related to job vulnerability due to illness-induced loss of occupational skills. This was reflected in specific language in the qualitative interviews.

Functioning sensory systems are vital in daily living (e.g., smelling dangerous gases, and spoiled food) as well as in fostering life’s qualities (smelling perfume, baked goods, wine) and critical to those in the food and wine profession. In a study where loss of taste and smell was the most predominant symptom, these patients are challenged by changes and limitations in their daily life and habits also noting the impact on relationships [24]. The effects of anosmia and ageusia from COVID-19 resulting in mental health challenges including depression, anxiety and low self-esteem were further documented in a narrative review by Javed et al. [25]. Additionally, Elkholi and colleagues [26] in a cross-sectional study with 487 COVID-19 patients with anosmia, found significant reductions in health-related quality of life with loss of smell directly affecting daily activities related to olfaction.

We believe the effects described in our cohort are more in tune to untoward quality of life effects following career-ending injuries in athletes and the subsequent changes in “life narrative” [27]. In fact, the qualitative language of our participants indicated an acute change in one life narrative with long-term fears of losing their lives intertwined with wine.

A notable proportion of respondents attempted to retain their senses, often seeking advice from colleagues and the internet. The majority reported some degree of success which is corroborated by literature specific to post-viral anosmia [28]. Respondents highlighted the lack of health information and options for promoting sensory health during their episodes. In fact, many respondents noted this drove their desire to participate in the study. As a signal symptom of COVID-19 infection, there has been a renewed interest in mechanism and treatment. Our data indicate the need for policy to improve access to patient evaluation and innovative approaches to therapy.

Most wine professionals undergo a systematic approach to the evaluation of wine involving sight, aroma, taste, mouthfeel and related attributes, e.g., finish or length. The validity of our findings relative to the science of wine assessment was evident by the fact that many participants who were affected with viral-induced taste and smell alterations retained their ability to evaluate the components of assessment known as mouthfeel. Somewhat independent of taste and aroma, mouthfeel relates to ethanol, and tannins with described feelings of astringency, body, burn, and viscosity. The tactile sensing papillae detect changes in salivary film from wine via the trigeminal nerve [29] somewhat distinct from taste and olfaction [30]. The viral-induced impairments in olfaction, for example, have their effects anatomically remote from the oral cavity where epithelial support cells of olfactory neurons are likely impaired [3].

## Strengths and limitations

A major study strength included the formation of an expert advisory group; this ensured that the study was practically relevant and reflective of wine professionals with lived experience of a COVID-19 infection. Other strengths included the international sampling approach, and the sequential explanatory mixed methods design which used qualitative results to explain the initial quantitative results in greater depth [15]. Limitations included the smaller quantitative sample size (n=199) which limits the generalizability and potential selection bias given the purposive sampling technique. Moreover, there is also the risk of recall bias from the use of self-reported data, especially among participants reporting sensory changes from infections that occurred 1–2 years prior as the exact duration and detail of sensory loss may only be approximate. Future research should consider exploring this topic using retrospective clinical records, if available.

## Conclusion

The present study describes the impact of sensory impairment in wine professionals following a COVID-19 infection. The quantitative findings provide a descriptive overview of the sensory impacts, while the qualitative findings show a deeper understanding into the emotional and professional toll wine professionals endured. Overall, the study provided a holistic picture of how COVID-19 affected wine professionals, not only in terms of sensory loss but also in their professional identity and well-being. The findings highlight a need for tailored support for wine professionals impacted by a COVID-19 infection. This might include interventions that focus on sensory rehabilitation (e.g., sensory retraining programs) as well as mental health and well-being supports that address the challenges associated with sensory loss. Future research should further uncover the sensory impact of COVID-19 across sensory-dependent professionals, in addition to the effectiveness of sensory retraining techniques and emerging pharmaceutical therapies [31].

## Supporting information

S1 File Quantitative Survey. (PDF)
