# Supplementary material for: Sensory effects of COVID-19 in wine professionals
Source: PLoS One. 2025 Apr 25;20(4):e0321502. doi: 10.1371/journal.pone.0321502 (PMC12027222; doi:10.1371/journal.pone.0321502)
Supplement: S1 File — (PDF) [file pone.0321502.s001.pdf]

Sensory Effects of COVID-19 in Wine Professionals

Title: The Effect of COVID-19 Infection on the Sensory Evaluation of Wine Professionals

Research Investigators: Dr. Theodore J. Witek, Jr. DrPH; Natasha Sheikhan, MPH, MHS; Allan Tran, MSc.

Investigators' Affiliations: Dalla Lana School of Public Health; Institute of Health, Policy, Management and Evaluation, University of Toronto.

The University of Toronto's Research Ethics Board (REB) has reviewed this study. If you have any questions about the rights of the participant, you can contact the Research Oversight and Compliance Office-Human Ethics Program at ethics.review@utoronto.ca or (416)-946-3273.

Participants may print out this information sheet and consent form for their own personal records.

INTRODUCTION

You are invited to participate in a survey about sensory evaluation of wine, following an infection with COVID-19 virus. You are being contacted because you have some level of wine education or training, which involves an informed approach to wine tasting.

You most likely know that there are reports of COVID-19 infection affecting taste and smell. We are interested in your accurate assessment of questions related to your wine tasting sensory skills associated with COVID-19 infection.

You may also be offered the opportunity to participate in a one-on-one interview (via telephone, Zoom or Microsoft Teams) as well as perform a very basic smell identification test.

Thank you for your interest in this study. To begin, please click the attached Consent Letter for this study below.

Please read the attachment carefully and then return to this page to respond to the consent question below.

[Attachment: "Consent Letter .pdf"]

Do you agree to participate in the evaluation study? ◯ Yes ◯ No

Part 1. Demographic Questions

1. What gender do you identify as?
   - ◯ Man
   - ◯ Woman
   - ◯ Other
   - ◯ Prefer not to answer

1a. Please indicate what gender you identify as.

____

2. What is your age?
   - ◯ 19-29 years old
   - ◯ 30-39 years old
   - ◯ 40-49 years old
   - ◯ 50+ years old

3. Where do you currently reside?
   - ◯ Canada
   - ◯ United States
   - ◯ United Kingdom
   - ◯ New Zealand
   - ◯ Australia
   - ◯ Other

3a. Please indicate where you reside.

____

4. What describes your ethnicity?

- ☐ Asian - East (e.g., Chinese, Japanese, Korean)
- ☐ Asian - South (e.g., Indian, Pakistani, Sri Lankan)
- ☐ Asian - South East (e.g., Malaysian, Filipino, Vietnamese)
- ☐ Black - African (e.g., Ghanian, Kenyan, Somali)
- ☐ Black - Caribbean (e.g., Barbadian, Jamaican)
- ☐ Black - North American (e.g., Canadian, American)
- ☐ Indigenous (e.g., First Nations, Inuit, Metis)
- ☐ Indian - Caribbean (e.g., Guyanese with origins in India)
- ☐ Latin American (e.g., Argentinean, Chilean, Salvadoran)
- ☐ Middle Eastern (e.g., Egyptian, Iranian, Lebanese)
- ☐ White - European (e.g., English, Italian, Portuguese, Russian)
- ☐ White - North American (e.g., Canadian, American)
- ☐ Mixed heritage (e.g., Black - African & White - North American)
- ☐ Other

4a. Please indicate your ethnicity.

____

5. What level of wine qualifications do you hold?

- ◯ No formal qualifications; Minimal practical experience
- ◯ No formal qualifications, Extensive practical; experience
- ◯ Low-mid level qualification (e.g., WSET 1-3)
- ◯ High level formal qualification (e.g., MW, WSET Diploma)

6. How many years have you been a wine professional (Note: Please include the time spent in training):

____

7. Does most of your income come from the wine industry and/or hospitality?

- ◯ Yes
- ◯ No

Part 2. COVID-19 General Questions

1. Did you have a documented COVID-19 infection?

- ◯ Yes
- ◯ No

2. Have you had more than one COVID-19 infection?

- ◯ Yes
- ◯ No

3. Did you experience any COVID-19 symptoms?

- ◯ Yes
- ◯ No

Please use your first infection to answer the following questions.

3. How were you diagnosed with COVID-19?

- ◯ PCR test
- ◯ Rapid antigen test (at home or in pharmacy)
- ◯ Both
- ◯ Neither

4. What period of the pandemic was your first infection?	◯ Sometime during 2020<br>◯ Sometime during 2021<br>◯ Sometime during 2022
5. Did you see a health care professional for treatment of your COVID-19 symptoms?	◯ Yes<br>◯ No
6. How would you describe your COVID-19 symptoms overall?	◯ Mild<br>◯ Moderate<br>◯ Severe
6a. Were you hospitalized due to your COVID-19 symptoms?	◯ Yes<br>◯ No
7. Choose the very first COVID-19 symptom that you noticed.	◯ Fever<br>◯ Cough<br>◯ Tiredness<br>◯ Loss of taste or smell<br>◯ Sore throat<br>◯ Headache<br>◯ Aches and pains<br>◯ Other
7a. Please indicate your first COVID-19 symptom.	______________________________
8. Over the course of your acute illness period, which symptoms did you experience (please check off all that apply)?	☐ Fever<br>☐ Cough<br>☐ Tiredness<br>☐ Loss of taste<br>☐ Loss of smell<br>☐ Sore throat<br>☐ Headache<br>☐ Aches and pains<br>☐ Other
8a. Please indicate what other symptom(s) you experienced.	______________________________
9. After your COVID-19 infection, did you attempt to re-train your senses in any manner?	◯ Yes<br>◯ No
9a. If yes, was it:	◯ Successful<br>◯ Partially successful<br>◯ Unsuccessful
10. Did you experience any long-term symptoms after having a COVID-19 infection, also referred to as 'long COVID'?	◯ Yes<br>◯ No

10a. How long did your symptoms last?	◯ About 1 week<br>◯ 2 weeks<br>◯ 3 weeks<br>◯ 4 weeks<br>◯ Longer than 4 weeks<br>◯ Greater than 1 month but less than 2 months<br>◯ Greater than 2 months but less than 3 months<br>◯ Greater than 3 months but less than 6 months<br>◯ Greater than 6 months
11. Were you worried about potential effects on your ability to taste and identify wine?	◯ Not at all worried<br>◯ Somewhat worried<br>◯ Very worried
12. Did you discuss the potential of losing your taste and/or smell with a colleague or friend/family?	◯ Yes<br>◯ No
13. Would you describe your vaccination status as:	◯ Choose not to vaccinate<br>◯ Received 1 dose<br>◯ Received 2 doses, no boosters<br>◯ Received 2 doses and at least 1 booster dose

Part 3. Taste and Smell Questions

1. At any point during or after your COVID-19 infection, did you experience any changes in your sense of taste?	◯ No changes to taste<br>◯ Loss of taste<br>◯ Distorted sense of taste
1a. To what extent did you experience taste loss?	◯ Mild / minor reduction (e.g., I lost a bit of my sense to taste)<br>◯ Moderate reduction (e.g., I lost most but not all of my taste)<br>◯ Severe / pronounced reduction (e.g., I could not taste anything)
1b. To what extent did you experience taste distortion?	◯ Mild / minor reduction (e.g., My taste was a bit distorted)<br>◯ Moderate reduction (e.g., My taste was semi-distorted)<br>◯ Severe / pronounced reduction (e.g., My taste was completely distorted)
1c. If you had a loss of taste or distortion, how long did it last?	◯ About 1 week<br>◯ 2 weeks<br>◯ 3 weeks<br>◯ 4 weeks<br>◯ Longer than 4 weeks<br>◯ Greater than 1 month but less than 2 months<br>◯ Greater than 2 months but less than 3 months<br>◯ Greater than 3 months but less than 6 months<br>◯ Greater than 6 months
Are your symptoms still persisting?	◯ Yes<br>◯ No
Did you discuss your concern about losing your taste or experiencing taste distortion from COVID-19 infection with a colleague, friend or family?	◯ Yes<br>◯ No

2. At any point during or after your COVID-19 infection, did you experience any changes in your sense of smell?	◯ No changes to smell<br>◯ Loss of smell<br>◯ Distorted sense of smell
2a. To what extend did you experience smell loss?	◯ Mild / minor reduction (e.g., I lost a bit of my sense to smell)<br>◯ Moderate reduction (e.g., I lost most but not all of my smell)<br>◯ Severe / pronounced reduction (e.g., I could not smell anything)
2b. To what extend did you experience smell distortion?	◯ Mild / minor reduction (e.g., My smell was a bit distorted)<br>◯ Moderate reduction (e.g., My smell was semi-distorted)<br>◯ Severe / pronounced reduction (e.g., My smell was completely distorted)
2c. If you had a loss of smell or distortion, how long did it last?	◯ About 1 week<br>◯ 2 weeks<br>◯ 3 weeks<br>◯ 4 weeks<br>◯ Longer than 4 weeks<br>◯ Greater than 1 month but less than 2 months<br>◯ Greater than 2 months but less than 3 months<br>◯ Greater than 3 months but less than 6 months<br>◯ Greater than 6 months
Are your symptoms still persisting?	◯ Yes<br>◯ No
Did you discuss your concern about losing your smell or experiencing smell distortion from COVID-19 infection with a colleague, friend or family?	◯ Yes<br>◯ No
2a. If you did not experience any effects on your smell or taste, were you:	◯ Not worried about any potential effects on smell or taste on your wine profession<br>◯ Slightly worried about any potential effects on smell or taste on your wine profession<br>◯ Very worried about any potential effects on smell or taste on your wine profession
2b. Did you discuss your concern about potentially losing your taste or smell from COVID-19 Infection with a colleague or friend, or family?	◯ Yes<br>◯ No

Part 4. Impact on professional life

1. How did your episode of lost taste and/or smell impact your involvement in your wine profession?	◯ Not at all<br>◯ Somewhat<br>◯ Significantly
2. Did you require time away from profession specifically related to your inability to taste and/or smell?	◯ Yes<br>◯ No

3. How has the loss of taste and/or smell impacted your overall quality of life

◯ None
◯ Minor
◯ Moderate
◯ Severe

4. You likely take a systematic approach to tasting wine. Check the appropriate box on the impact of COVID-19 on evaluating:

	None	Some	A lot
Flavour Profile	◯	◯	◯
Taste Profile	◯	◯	◯
Alcohol Level	◯	◯	◯
Acid Level	◯	◯	◯
Tannin Level	◯	◯	◯
Finish	◯	◯	◯

Did you know any colleagues in the wine profession who lost or had an alteration in their taste or smell following a COVID-19 infection?

◯ Yes
◯ No

Please forward them the following link: https://redcap.utoronto.ca/surveys/?s=KWT49FCJE47DR4H7 via email and please ensure to CC our email address: wine.covid@gmail.com.

We need to know that real people are actually doing the survey. Please choose '6' as your answer and continue to the next question.

◯ 1 ◯ 2 ◯ 3 ◯ 4
◯ 5 ◯ 6 ◯ 7 ◯ 8
◯ 9

Given that COVID-19 infection caused the loss or alteration of your smell and/or taste, you are invited to participate in a brief remote (telephone, Zoom or Microsoft Teams) interview to further explore your experience. These interviews will be approximately 20 minutes in length. You will be asked if a recording is possible and we will only proceed with recording with your consent. Otherwise, we will simply take notes.

While there are no risks to this survey, you will benefit the scientific and wine community in understanding the effects of the COVID-19 infection on sensory effects.

Click here on the following link to schedule an interview.
